# An HIV Diagnostic Testing Algorithm Using the cobas HIV-1/HIV-2 Qualitative Assay for HIV Type Differentiation and Confirmation

**DOI:** 10.1128/JCM.03030-20

**Published:** 2021-06-18

**Authors:** Dana Duncan, John Duncan, Bastian Kramer, Alex Y. Nilsson, Betiel Haile, Ann Butcher, Shikha Chugh, Paul Baum, Grace M. Aldrovandi, Stephen Young, Ann K. Avery, Karen Tashima, Alexandra Valsamakis, Joseph D. Yao, Ming Chang, Robert W. Coombs

**Affiliations:** a Roche Molecular Systems, Pleasanton, California, USA; b Roche Molecular Systems, Basel, Switzerland; c Department of Pediatrics, University of California, Los Angeles, Los Angeles, California, USA; d TriCore Reference Laboratories, Albuquerque, New Mexico, USA; e MetroHealth, Cleveland, Ohio, USA; f The Miriam Hospital, Providence, Rhode Island, USA; g Division of Clinical Microbiology, Department of Laboratory Medicine and Pathology, Mayo Clinic, Rochester, Minnesota, USA; h Department of Laboratory Medicine and Pathology, University of Washington, Seattle, Washington, USA; i Department of Medicine, University of Washington, Seattle, Washington, USA; UNC School of Medicine

**Keywords:** HIV-1, HIV-2, diagnosis, PCR, testing algorithm

## Abstract

Human immunodeficiency virus types 1 and 2 (HIV-1 and HIV-2) diagnostic testing algorithms recommended by the Centers for Disease Control involve up to three tests and rely mostly on detection of viral antigen and host antibody responses. HIV-1 p24 antigen/HIV-1/HIV-2 antibody-reactive specimens are confirmed with an immunochromatographic HIV-1/HIV-2 antibody differentiation assay, and negative or indeterminate results from the differentiation assay are resolved by an HIV-1-specific nucleic acid amplification test (NAT). The performance of a proposed alternative algorithm using the cobas HIV-1/HIV-2 qualitative NAT as the differentiation assay was evaluated in subjects known to be infected with HIV-1 (*n* = 876) or HIV-2 (*n* = 139), at low (*n* = 6,017) or high (*n* = 1,020) risk of HIV-1 infection, or at high-risk for HIV-2 infection (*n* = 498) (study A). The performance of the cobas HIV-1/HIV-2 qualitative test was also evaluated by comparison to an HIV-1 or HIV-2 alternative NAT (study B). The HIV-1 and HIV-2 overall percent agreements (OPA) in study A ranged from 95% to 100% in all groups. The positive percent agreements (PPA) for HIV-1 and HIV-2 were 100% (876/876) and 99.4% (167/168), respectively, for known positive groups. The negative percent agreement in the HIV low-risk group was 100% for both HIV-1 and HIV-2. In study B, the HIV-1 and HIV-2 OPA ranged from 99% to 100% in all groups evaluated (*n* = 183 to 1,030), and the PPA for HIV-1 and HIV-2 were 100% and 99.5%, respectively, for known positive groups. The cobas HIV-1/HIV-2 qualitative assay can discriminate between HIV-1 and HIV-2 based on HIV RNA and can be included in an alternative diagnostic algorithm for HIV.

## INTRODUCTION

Human immunodeficiency virus type 1 (HIV-1) is the predominant cause of AIDS worldwide, with approximately 38 million people infected (https://www.unaids.org/en/resources/documents/2019/2019-UNAIDS-data). HIV-2, mainly found in West Africa, can also cause AIDS, and between 1 and 2 million people are infected worldwide (1). The distinction between HIV-1 and HIV-2 is important for several reasons, including (i) HIV-2 is less virulent than HIV-1, with lower viral loads and slower progression to opportunistic infections; (ii) to select the appropriate type of viral load test to use for treatment monitoring; and (iii) to avoid use of some antiretroviral drugs in persons infected with HIV-2, particularly nonnucleotide reverse transcriptase inhibitors and some protease inhibitors, that are not effective against HIV-2 (1–3). Coinfection with both HIV-1 and HIV-2 viruses is also possible; approximately 1% of those with HIV-1 infection in West Africa are coinfected with HIV-2. Coinfection has no effect on the rate of progression to AIDS but does complicate viral load monitoring and antiviral treatment (1, 2). As HIV-2 has spread outside West Africa, the U.S. Centers for Disease Control and Prevention (CDC) algorithm for HIV diagnosis includes testing for HIV-2 antibodies to enable differentiation from HIV-1 infections (4, 5). The European guidelines on HIV testing and several European country guidelines also recommend differentiation between HIV-1 and HIV-2 (6–8).

**FIG 1:**
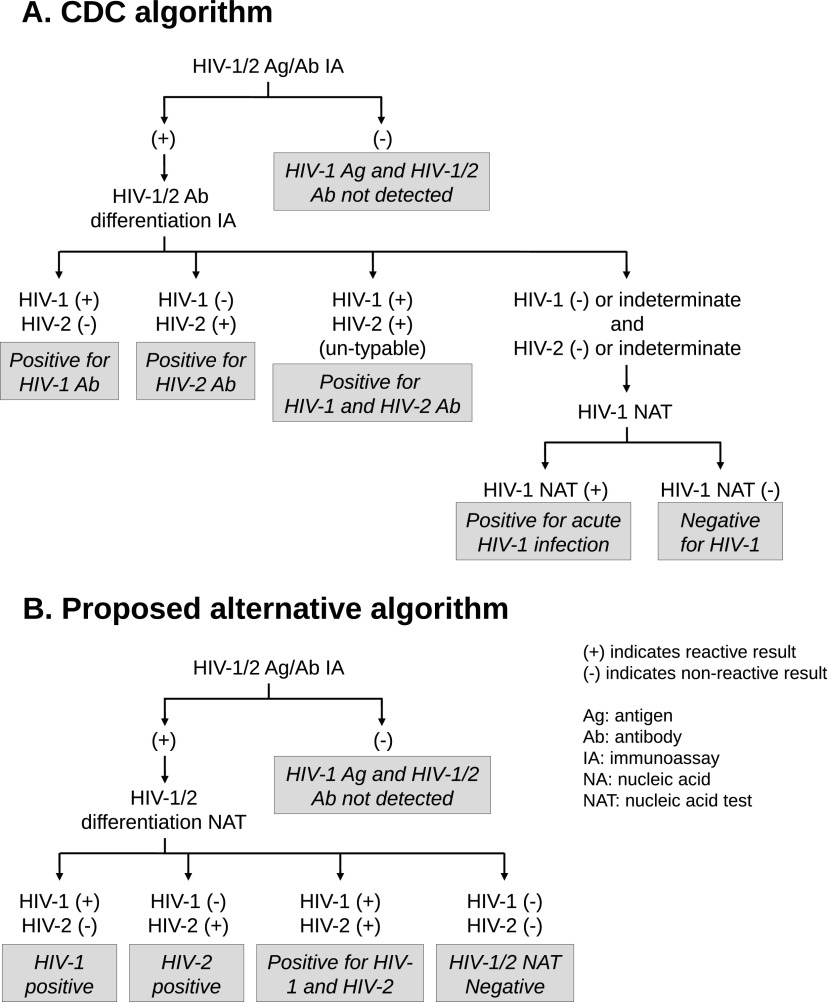
Comparison of HIV-1/HIV-2 testing algorithms. (A) CDC HIV testing algorithm (9). Ag, antigen; Ab, antibody; IA, immunoassay; NA, nucleic acid; NAT, nucleic acid test. In this study, the HIV-1/2 Ag/Ab IA was Abbott Architect, the HIV-1/2 Ab differentiation IA was the Bio-Rad Geenius HIV1/2 supplemental assay, and the HIV-1 NAT is the Aptima HIV-1 RNA qualitative assay. (B) Proposed alternative algorithm that uses an HIV-1/2 differentiation NAT (cobas HIV-1/HIV-2 qualitative NAT for use on the cobas 6800/8800 systems) instead of Geenius.

The current HIV diagnostic testing algorithm recommended by the CDC (CDC algorithm) includes use of a screening antigen/antibody immunoassay followed by an immunoassay to differentiate between HIV-1 and HIV-2 with a nucleic acid amplification test (NAT) if needed to resolve discordant or indeterminate serology results (Fig. 1A) (4, 9). Currently, two HIV-1/HIV-2 differentiation immunoassays are approved by the U.S. Food and Drug Administration (FDA), the Geenius HIV1/2 supplemental assay (Geenius; Bio-Rad Laboratories, Redmond, WA) (10–12) and the VioOne HIV Profile supplemental assay (Avioq, Research Triangle Park, NC) (13). Differentiating HIV-1 and HIV-2 using the Geenius assay is challenging due to the generation of HIV-1-indeterminate, HIV-2-indeterminate, HIV-indeterminate, and HIV-positive untypeable results which require subsequent nucleic acid testing to resolve (14–16). The Geenius assay detects the gp140 and gp36 HIV-2 antigens in separate bands, and reactivity in only one band leads to HIV-indeterminate or HIV-2-indeterminate results depending on the remaining HIV-1-specific banding patterns. Bio-Rad has performed a software upgrade (v1.3) to raise the cutoff value of the gp140 band and reduce the number of indeterminate results (9). Alternative testing algorithms have also been proposed to mitigate the challenges of indeterminate results, including using a quantitative HIV-1 NAT as the second step of the algorithm (16).

This evaluation explores the use of a qualitative NAT that can differentiate HIV-1 and HIV-2 RNA, the cobas HIV-1/HIV-2 qualitative NAT for use on the cobas 6800/8800 systems (cobas HIV-1/HIV-2 Qual; Roche Molecular Systems, Branchburg, NJ), a CE-marked and FDA-approved *in vitro* diagnostic assay (17). The evaluation is divided into two parts, study A and study B. In study A, the performance of the CDC algorithm was compared to a proposed alternative algorithm using the cobas HIV-1/HIV-2 Qual instead of an immunoassay for HIV-1/2 differentiation. In study B, the performance of the cobas HIV-1/HIV-2 Qual was compared directly to an FDA-approved HIV-1 qualitative NAT (Aptima HIV-1 RNA qualitative assay [Aptima Qual]; Hologic Inc., San Diego, CA) (18) and a laboratory-developed HIV-2 quantitative NAT (LDT) in use by the University of Washington reference laboratory (University of Washington HIV-2 Plasma RNA quantitative assay [UW HIV-2]; University of Washington, Seattle, WA) (19).

## MATERIALS AND METHODS

### Ethics statement.

The study protocol was approved by local institutional review boards (IRB) in accordance with FDA and local regulatory requirements before the start of the study.

### Specimens and study populations.

In study A, the performance of the CDC algorithm was compared to a proposed alternative algorithm, including the cobas HIV-1/HIV-2 Qual test in subjects known to be infected with HIV-1 or HIV-2, subjects at high risk for HIV-1 or HIV-2 infection, and subjects at low risk for HIV infection.

In study B, the performance of the cobas HIV-1/HIV-2 Qual was compared to that of Aptima Qual and UW HIV-2 using specimens from subjects known to be infected with HIV-1 or HIV-2, subjects at high risk for HIV-1 infection, and specimens with serology-discordant results (e.g., antibody/antigen [Ab/Ag] positive and HIV-1/HIV-2 differentiation assay negative or indeterminate). The algorithms for study A are shown in Fig. 1.

For both studies, specimens with insufficient volume to perform NAT testing for both targets were included in agreement analysis for only one target.

**FIG 2:**
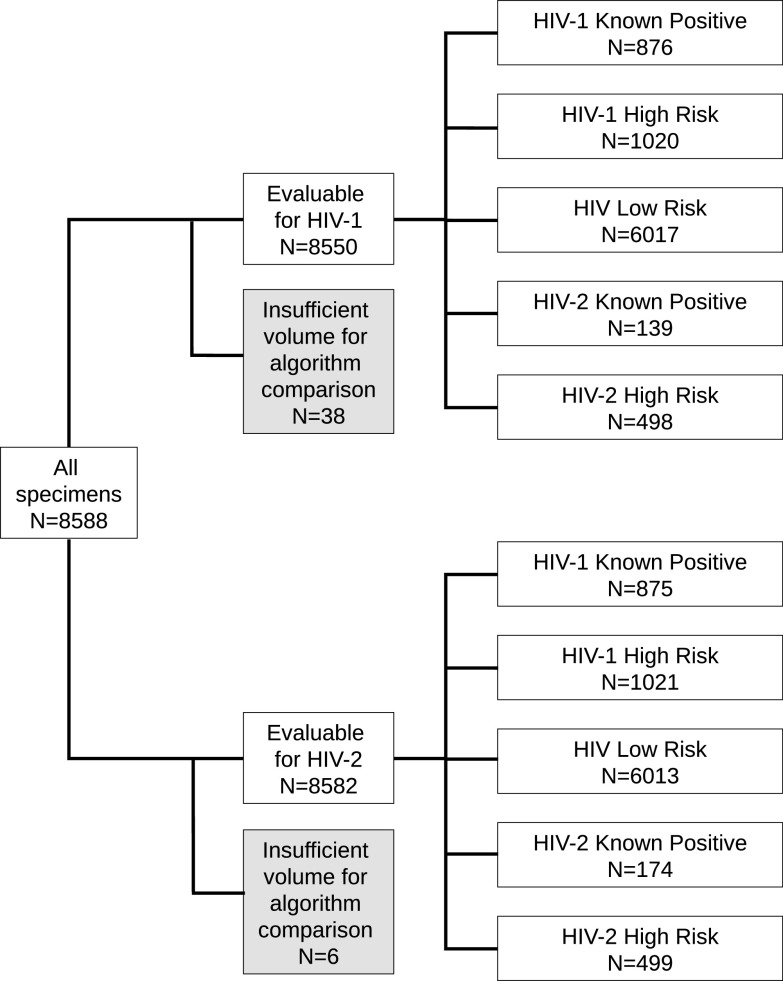
Specimens included in agreement analyses between CDC and proposed alternative HIV testing algorithm (study A).

**FIG 3:**
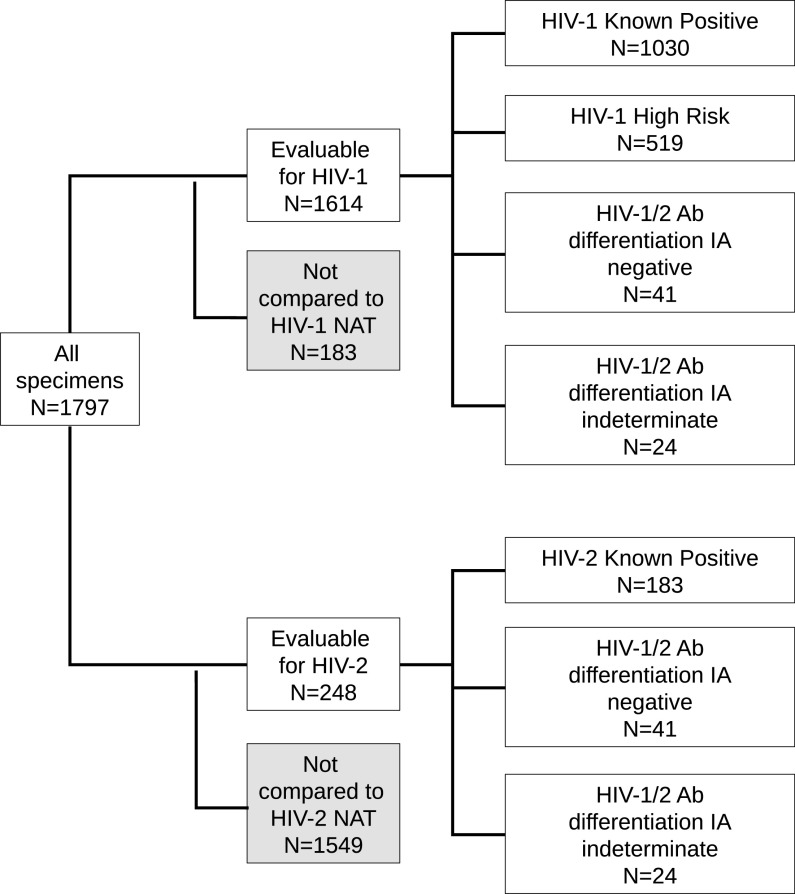
Specimens included in agreement analyses between cobas HIV-1/HIV-2 Qual and alternative NAT assays (study B).

The criteria used to define the different groups of subjects are detailed below and summarized in Table S1 in the supplemental material. Studies A and B used the same subject group definitions but different specimens; the numbers of specimens that were evaluable for each comparison by HIV target for each group are displayed in Fig. 2 (study A) and Fig. 3 (study B; see Table S2 for details of overlap between studies).

Deidentified specimens (plasma and serum) were obtained from a combination of commercial suppliers and clinical laboratories. The specimens were collected from 2007 to 2019 and stored frozen at −20°C or below until the time of testing. In study A, the majority of specimens with known country of origin were from the United States (83%) (Table S3); in study B, 46% were from the United States (Table S4). The HIV-1 non-B subtype, HIV-2 high-risk, and HIV-2-positive samples were collected primarily from African countries with a high prevalence of HIV-2 infection. Pregnant women represented 23.1% and 0.8% of the HIV-1 high-risk and HIV low-risk specimens, respectively, and children (<18 years) represented 19.6% and 0.9% of the HIV-1 high-risk and HIV low-risk specimens, respectively. In the HIV-1 known positive group, 35% of specimens were from African countries where non-B subtypes are prevalent. For the HIV-1 and HIV-2 known positive populations, the HIV load was determined by the vendor or source laboratory using one of several HIV-1 quantitative assays or by UW HIV-2. Only HIV known positive samples with viral load greater than 100 copies/ml were included in the study since the clinical sensitivity of Aptima Qual was determined using samples with viral RNA concentrations over this level (18). The HIV status of the specimens in the HIV-1 or HIV-2 high-risk and HIV low-risk groups was not known prior to inclusion. All specimens from subjects at high risk for HIV-1 or HIV-2 infection were from subjects with at least 1 risk factor for HIV infection (see Table S1), and all specimens from subjects at high risk for HIV-2 infection were from subjects living in Cote d’Ivoire, where HIV-2 is endemic. Specimens from subjects at low risk for HIV infection were collected from healthy blood donors and routine clinical screening in geographic areas with less than 1% HIV prevalence.

In study B, due to limited sample volume, 45 of 65 specimens with known serology-discordant results (e.g., Ab/Ag positive and HIV-1/HIV-2 differentiation assay negative or indeterminate) were diluted up to 4-fold with HIV-1-negative plasma from a single donor to obtain the required volume for testing with cobas HIV-1/2 Qual and the comparator NAT. No other specimens were diluted.

### HIV testing.

All diagnostic tests were performed according to the manufacturer’s instructions at one of the following six test sites: Cenetron Diagnostics (Austin, TX), Q2 Solutions (Valencia, CA), Center for Disease Detection (San Antonio, TX), TriCore Reference Laboratories (Albuquerque, NM), University of Washington Retrovirology Laboratory (Seattle, WA), or Roche Molecular Systems (Pleasanton, CA). All testing sites except for Roche Molecular Systems are Clinical Laboratory Improvement Amendment (CLIA)- and College of American Pathologists (CAP)-certified laboratories.

Following the CDC algorithm (Fig. 1A), the Architect HIV Ag/Ab combo test (Architect; Abbott Laboratories, Abbott Park, IL) was used as the screening immunoassay, the Geenius assay was used as the differentiation assay, Aptima Qual was used as the HIV-1 NAT, and UW HIV-2 (19) was used as the HIV-2 NAT. The performance of the CDC algorithm was compared to a proposed alternative algorithm, which includes the cobas HIV-1/HIV-2 Qual test (Fig. 1B). In cases in which there was a discrepancy between the results from the cobas HIV-1/HIV-2 Qual and Aptima Qual tests or between the CDC and proposed alternative algorithm, additional testing with the Aptima HIV-1 Quant Dx Assay (Aptima Quant; Hologic Inc., San Diego, CA) or UW HIV-2 was performed when there was sufficient specimen volume.

The limit of detection (LOD) of HIV-1 for plasma with the Aptima Quant assay is 12 copies/ml, and the Aptima Qual assay is expected to give positive results in 98.5% of replicate tests of a specimen with 30 copies/ml (18, 20). Serum specimens with viral load results reported here indicate only that these specimens were reactive for HIV-1 RNA. An Aptima Quant result of detected but below 30 copies/ml in serum specimens was classified as HIV reactive but below the lower limit of quantitation. The LOD for HIV-2 with the UW HIV-2 test is 8 copies/ml (19). The LOD for the cobas HIV-1/HIV-2 Qual for HIV-1 group M in plasma is 12 copies/ml and for HIV-2 is 35 copies/ml (17).

### Data analysis.

All data analyses were performed using SAS/STAT software (Cary, NC). Specimens with sufficient sample volume for completion of the CDC algorithm, the cobas HIV-1/HIV-2 Qual test, and alternate NAT (if required) were included in statistical analyses. Positive, negative, and overall percent agreements (PPA, NPA, and OPA) and associated 2-sided 95% Clopper-Pearson exact confidence intervals (CIs) for comparisons between assays and algorithms were calculated for each viral target (HIV-1 and HIV-2).

## RESULTS

### Study A: algorithm comparison for HIV-1.

A total of 8,550 specimens were included in study A (Fig. 2). These specimens were from subjects known to be infected with HIV-1 (*n* = 876) or HIV-2 (*n* = 139), subjects at high risk for HIV-1 (*n* = 1,020) or HIV-2 (*n* = 498) infection, and subjects at low risk for HIV infection (*n* = 6,017). The percent agreements for HIV-1 between the proposed alternative algorithm and the CDC HIV testing algorithm for each population are shown in Table 1.

**TABLE 1:** Percent agreement for HIV-1 between a proposed alternative algorithm and the CDC HIV testing algorithm (study A)

Study population	Positive percent agreement		Negative percent agreement		Overall percent agreement	
	% (no. of specimens/total no. of specimens)	95% CI^*g*^ (%)	% (no. of specimens/total no. of specimens)	95% CI (%)	% (no. of specimens/total no. of specimens)	95% CI (%)
HIV-1 known positive	100 (876/876)	99.6–100	NA		100 (876/876)	99.6–100
HIV-1 high risk	58 (29/50)^*a*^	43.2–71.8	100 (970/970)	99.6–100	97.9 (999/1,020)^*a*^	96.9–98.7
HIV low risk	66.7 (10/15)^*b*^	38.4–88.2	100 (6,002/6,002)	99.9–100	99.9 (6,012/6,017)^*b*^	99.8–99.99
HIV-2 known positive	14.3 (1/7)^*c*^	0.4–57.9	99.3 (137/138)^*d*^	96.0–99.98	95.2 (138/145)^*c*^^,^^*d*^	90.3–98.0
HIV-2 high risk	77.7 (80/103)^*e*^	68.4–85.3	99.7 (394/395)^*f*^	98.6–99.994	95.2 (474/498)^*e*^^,^^*f*^	92.9–96.9

a Nineteen discordant specimens had target not detected results, and two had results of <30 copies/ml on Aptima Quant.

b Three discordant specimens had TND results, and two plasma specimens had results of <30 copies/ml on Aptima Quant.

c Six discordant specimens had HIV-2 viral loads ranging from 197 to 2,190 copies/ml by UW HIV-2 and HIV-1-positive results by Geenius in this HIV-2 known positive population.

d One discordant specimen had an HIV-1 viral load of 5,954 copies/ml on Aptima Quant.

e Eighteen discordant specimens were TND, 4 plasma specimens had results of <30 copies/ml, and 1 serum specimen was reactive on Aptima Qual (<30 copies/ml).

f One discordant specimen had an HIV-1 viral load of 111.5 copies/ml on Aptima Quant.

g CI, confidence interval; NA, not applicable for this population.

In subjects known to be infected with HIV-1, the HIV-1 PPA was 100% (876/876; 95% CI, 99.6% to 100%), and in subjects at high risk for HIV-1 infection, the HIV-1 PPA was 58.0% (29/50; 95% CI, 43.2% to 71.8%). All 21 specimens with discordant results from subjects at high risk for HIV-1 infection were HIV-1 positive by Geenius. Of these 21 specimens, 19 were HIV-1 negative (target not detected [TND]) on Aptima Quant, and 2 were reactive but had viral loads of less than 30 copies/ml. All 21 specimens were from pregnant women.

In subjects at low risk for HIV infection, the HIV-1 PPA was 66.7% (10/15; 95% CI, 38.4% to 88.2%). All five discordant specimens were HIV-1 positive by Geenius. Three of the five specimens were HIV-1 RNA negative (TND) on Aptima Quant, and two had HIV-1 viral load below 30 copies/ml. The HIV-1 NPA was 100% (6,002/6,002; 95% CI, 99.9% to 100%).

All but one specimen from subjects known to be infected with HIV-2 had negative HIV-1 test results, leading to an HIV-1 NPA of 99.3% (137/138; 95% CI, 96.0% to 99.98%). The discordant specimen had a Geenius assay result of HIV-2 positive with HIV-1 cross-reactivity and had an HIV-1 viral load of 5,954 copies/ml. The HIV-1 PPA was 14.3% (1/7; 95% CI, 0.4% to 57.9%) in subjects known to be infected with HIV-2 and was due to six specimens with apparent false-HIV-1-positive Geenius results. These six specimens had HIV-2 viral loads ranging from 197 to 2,190 copies/ml on UW HIV-2.

The HIV-1 PPA was 77.7% (80/103; 95% CI, 68.4% to 85.3%) in subjects at high risk for HIV-2 infection. Of the 23 discordant specimens, 21 were HIV-1 positive, and 2 were HIV positive but untypeable by Geenius. Eighteen of these 23 specimens were HIV-1 NAT negative by Aptima Quant, and 5 had viral load below 30 copies/ml. The HIV-1 NPA was 99.7% (394/395; 95% CI, 98.6% to 99.994%). The discordant specimen had a Geenius assay result of HIV-2 positive with HIV-1 cross-reactivity and HIV-1 viral load of 112 copies/ml.

### Study A: algorithm comparison for HIV-2.

A total of 8,582 specimens were included in the comparison between the proposed alternative algorithm and the CDC algorithm for HIV-2 (Fig. 2). These specimens were from subjects known to be infected with HIV-1 (*n* = 875) or HIV-2 (*n* = 174), subjects at high risk for HIV-1 (*n* = 1,021) or HIV-2 (*n* = 499) infection, and subjects at low risk for HIV infection (*n* = 6,013). The percent agreements for HIV-2 between the proposed alternative algorithm and the CDC HIV testing algorithm for each population are shown in Table 2.

**TABLE 2:** Percent agreement for HIV-2 between a proposed alternative algorithm and the CDC HIV testing algorithm (study A)

Study population	Positive percent agreement		Negative percent agreement		Overall percent agreement	
	% (no. of specimens/total no. of specimens)	95% CI^*e*^ (%)	% (no. of specimens/total no. of specimens)	95% CI (%)	% (no. of specimens/total no. of specimens)	95% CI (%)
HIV-2 known positive	99.4 (167/168)^*a*^	96.7–99.98	0 (0/6)^*b*^	0–45.9	96.0 (167/174)^*a*^^,^^*b*^	91.9–98.4
HIV-2 high risk	36.4 (4/11)^*c*^	10.9–69.2	100 (488/488)	99.2–100	98.6 (492/499)^*c*^	97.1–99.4
HIV low risk	NA		100 (6,013/6,013)	99.9–100	100 (6,013/6,013)	99.9–100
HIV-1 known positive	NA		99.9 (874/875)^*d*^	99.4–99.997	99.9 (874/875)^*d*^	99.4–99.997
HIV-1 high risk	NA		100 (1,021/1,021)	99.6–100	100 (1,021/1,021)	99.6–100

a One discordant specimen had an HIV-2 viral load of 198 copies/ml by UW HIV-2.

b Six discordant specimens had HIV-2 viral loads ranging from 197 to 2,190 copies/ml by UW HIV-2.

c Four discordant specimens had TND results and 3 had results of <10 copies/ml by UW HIV-2.

d One discordant specimen had TND results by UW HIV-2.

e CI, confidence interval; NA, not applicable for this population.

In subjects known to be infected with HIV-2, the HIV-2 PPA was 99.4% (167/168; 95% CI, 96.7% to 99.98%). The HIV-2-discordant specimen had an HIV-2 viral load of 198 copies/ml by UW HIV-2. The HIV-2 NPA was 0%, based on six specimens from subjects known to be infected with HIV-2 that were HIV-2 positive by the alternative algorithm and HIV-2 negative by the CDC algorithm, which had HIV-1-positive results on Geenius.

The HIV-2 PPA was 36.4% (4/11; 95% CI, 10.9% to 69.2%) in subjects at high risk for HIV-2 infection. Of the seven specimens with HIV-2-negative alternative algorithm results and HIV-2-positive CDC algorithm results, three were HIV-2 positive, three were HIV positive but untypeable, and one was HIV-2 positive with HIV-1 cross-reactivity by Geenius. UW HIV-2 testing of these seven specimens revealed that four specimens were HIV-2 RNA negative (TND), and three had HIV-2 viral load below 10 copies/ml.

In subjects at low risk for HIV infection, all HIV-2 test results were negative, resulting in an HIV-2 NPA of 100% (6,013/6,013; 95% CI, 99.9% to 100%).

In subjects known to be infected with HIV-1, the HIV-2 NPA was 99.9% (874/875; 95% CI, 99.4% to 99.997%). Two specimens were not included because of insufficient volume to perform HIV-2 NAT to resolve serology-discordant results. All but one specimen were negative for HIV-2 by both algorithms. The HIV-2-discordant specimen was HIV-2 positive by the alternative algorithm and HIV-2 negative with the CDC algorithm but was HIV-2 RNA negative (TND) by UW HIV-2. It was also HIV-1 positive according to both algorithms. All HIV-2 test results were negative in subjects at high risk for HIV-1 infection; thus, the HIV-2 NPA was 100% (1,021/1,021; 95% CI, 99.6% to 100%).

### Study A: algorithm comparison for HIV-1/HIV-2 dual infections.

There were 36 specimens classified as dual infections by one of the algorithms, including one case classified as dual infection by both algorithms. NAT testing, required to confirm dual infections, could not be performed for 29 of the 36 specimens due to insufficient specimen volume. Of the seven specimens with sufficient volume for investigation, three (subjects 1 to 3) were classified as potentially dually infected according to the CDC algorithm only (Table 3), three (subjects 4 to 6) were classified as dually infected according to the proposed alternative algorithm only, and one (subject 7) was classified as dually infected by both algorithms (HIV-1 viral load of 59,106 copies/ml and an HIV-2 viral load of 210 copies/ml; Table 3). Of the four specimens classified as potentially dually infected according to the CDC algorithm, Geenius test results were HIV positive but untypeable, indicating that HIV-1 and/or HIV-2 infection was present; only subject 7 had dual infection confirmed by HIV-1 viral load testing and UW HIV-2 NAT. Of the three specimens where dual infection was not confirmed, two (subjects 1 and 2) were HIV-1 negative on Aptima Qual and HIV-2 negative on UW HIV-2 NAT, and one (subject 3) was HIV-1 negative on Aptima Qual and HIV-2 positive (<10 copies/ml) on UW HIV-2 NAT. Of the four specimens classified as dually infected using the proposed alternative algorithm because of dually reactive cobas HIV-1/HIV-2 Qual test results, three (subjects 4, 5, and 7) were confirmed dual infections by HIV-1 viral load testing and UW HIV-2 NAT. The one specimen (subject 6) where dual infection was not confirmed was HIV-1 positive by Geenius and HIV-2 negative by HIV-2 NAT.

**TABLE 3:** Test results for subjects potentially dually infected with HIV-1 and HIV-2

Subject ID	Architect result	Geenius result	Cobas HIV-1/HIV-2 qual result	Aptima Quant HIV-1 viral load (copies/ml)^*a*^	UW HIV-2 viral load (copies/ml)	CDC algorithm	Alternative algorithm
1	Positive	HIV positive, untypeable	HIV-1 reactive	TND	TND	Positive for HIV-1 and HIV-2 Ab	HIV-1 positive
2	Positive	HIV positive, untypeable	Nonreactive	TND	TND	Positive for HIV-1 and HIV-2 Ab	HIV Ab positive, NAT negative
3	Positive	HIV positive, untypeable	Nonreactive	TND	<10	Positive for HIV-1 and HIV-2 Ab	HIV Ab positive, NAT negative
4	Positive	HIV-2 positive with HIV-1 cross-reactivity	HIV-1 and HIV-2 reactive	5,954	329	Positive for HIV-2 Ab	Positive for HIV-1 and HIV-2
5	Positive	HIV-2 positive with HIV-1 cross-reactivity	HIV-1 and HIV-2 reactive	112	28	Positive for HIV-2 Ab	Positive for HIV-1 and HIV-2
6	Positive	HIV-1 positive	HIV-1 and HIV-2 reactive	QNS	TND	Positive for HIV-1 Ab	Positive for HIV-1 and HIV-2
7	Positive	HIV positive, untypeable	HIV-1 and HIV-2 reactive	59,106	210	Positive for HIV-1 and HIV-2 Ab	Positive for HIV-1 and HIV-2

a TND, target not detected; QNS, quantity not sufficient.

### Study B: method comparison between cobas HIV-1/HIV-2 Qual and Alternative NAT assays.

A total of 1,797 specimens were included in the comparison between cobas HIV-1/HIV-2 Qual and alternative NAT assays (Fig. 3). These specimens were from subjects known to be infected with HIV-1 (*n* = 1,030) or HIV-2 (*n* = 183), subjects at high risk for HIV-1 (*n* = 519), and specimens with Ab/Ag-positive and HIV-1/HIV-2 differentiation assay negative (*n* = 41) or indeterminate (*n* = 22) results. Details of the percent agreement calculations between the two assays are shown in Tables S5 to S9 in the supplemental material.

In subjects known to be infected with HIV-1, the HIV-1 PPA and OPA of the cobas HIV-1/HIV-2 Qual test compared to Aptima Qual were 100% (1,029/1,029; 95% CI, 99.6% to 100%) and 99.9% (1,029/1,030; 95% CI, 99.5% to 99.998%), respectively (Table S5). All specimens were HIV-1 reactive by the cobas HIV-1/HIV-2 Qual test. One specimen (subtype K) was negative by Aptima Qual and had an HIV-1 viral load of 152 copies/ml with Aptima Quant.

The HIV-1 PPA and NPA in subjects at high risk for HIV-1 infection were 100% (5/5; 95% CI, 47.8% to 100%) and 100% (514/514; 95% CI, 99.3% to 100%), respectively (Table S6).

In subjects known to be infected with HIV-2, the HIV-2 PPA and OPA of the cobas HIV-1/HIV-2 Qual test compared to dichotomized positive/negative results from UW HIV-2 were both 99.5% (182/183; 95% CI, 97.0% to 99.99%; Table S7). The single discordant specimen had an HIV-2 viral load of 198 copies/ml and tested negative for HIV-2 on the cobas HIV-1/HIV-2 Qual test.

In specimens with Ab/Ag-positive (Architect) and Ab/Ag-negative Geenius results, the HIV-1 PPA between the cobas HIV-1/HIV-2 Qual test compared with Aptima Qual was 80% (4/5; 95% CI, 28.4% to 99.5%), and the HIV-1 NPA was 100% (36/36; 95% CI, 90.3% to 100%). The HIV-1 OPA was 97.6% (40/41; 95% CI, 87.1% to 99.9%; Table S8). The one discordant specimen was found to have an HIV-1 viral load of <30 copies/ml. HIV-2 reactivity was not observed with either assay. The HIV-2 NPA between the cobas HIV-1/HIV-2 Qual test compared with dichotomized positive/negative results from UW HIV-2 was 100% (41/41; 95% CI, 91.4% to 100%).

In specimens with Ab/Ag-positive (Architect) and indeterminate Geenius results, the HIV-1 PPA between the cobas HIV-1/HIV-2 Qual test and the Aptima Qual was 100% (5/5; 95% CI, 47.8% to 100%). The HIV-1 NPA between the cobas HIV-1/HIV-2 Qual test and the Aptima Qual was 94.7% (18/19; 95% CI, 74.0% to 99.9%; Table S9). One specimen was found to have an HIV-1 viral load of <30 copies/ml. The HIV-1 OPA between the cobas HIV-1/HIV-2 Qual test and the Aptima Qual was 95.8% (23/24; 95% CI, 78.9% to 99.9%). The HIV-2 PPA was not calculated, as HIV-2 reactivity was not observed on either assay. The HIV-2 NPA between the cobas HIV-1/HIV-2 Qual test and the dichotomized positive/negative results from UW HIV-2 was 100% (24/24; 95% CI, 85.8% to 100%).

## DISCUSSION

In this study, the performance of the current CDC HIV diagnostic algorithm was compared to a proposed alternative algorithm that uses cobas HIV-1/HIV-2 Qual, an HIV-1/HIV-2 qualitative NAT, instead of an immunoassay for HIV-1/HIV-2 differentiation. In persons known to be infected with HIV-1 or HIV-2, the PPA and OPA between algorithms were very high (>99% for HIV-1, >96% for HIV-2). Six discordant HIV-2 results were identified with positive results by cobas HIV-1/HIV-2 Qual results and confirmed as positive by alternative HIV-2 NAT testing (viral loads ranging from 197 to 2,190 copies/ml) but with HIV-1-positive and HIV-2-negative results by the CDC algorithm due to false-negative HIV-2 serology results. Using an HIV NAT as the differentiation assay may improve HIV-2 detection over current methods. Additionally, in two of three specimens confirmed as dual infections by NAT testing, the CDC algorithm misclassified them as HIV-2 monoinfections, suggesting that using a NAT for HIV-1/HIV-2 differentiation may also improve detection of dual infections over current methods.

In persons at low or high risk for HIV-1 or HIV-2 infection, for which neither HIV status nor viral load was known prior to study inclusion, the NPA was >99.7% for both targets, while the PPA was lower (36% to 78%). The primary reason for low PPA was positive serology results but undetectable HIV RNA. The discordant results were all HIV-1 or HIV-2 negative by cobas HIV-1/HIV-2 Qual and confirmed negative by alternative HIV-1 and HIV-2 NAT testing. Possible explanations for the discordant results include suppression of viral load by undisclosed antiretroviral drug use by the subject, such as antiretroviral therapy (ART) or preexposure prophylaxis (PrEP), suppression by the host immune response as seen in elite controllers (21), or vaccine-induced seropositivity/reactivity since prior participation in an HIV vaccine trial was not known (22). Additionally, directly comparing cobas HIV-1/HIV-2 Qual to an alternative HIV-1 (Aptima Qual) or HIV-2 (UW HIV-2 LDT) NAT in persons with known HIV infection, the PPA and OPA were very high (>99.5% for both targets) with the one discordant result positive by cobas HIV-1/HIV-2 Qual and confirmed by alternative NAT testing, indicating a false-negative Aptima Qual result. In subjects at high risk for HIV-1 infection, the PPA, NPA, and OPA were all 100%. The challenges associated with use of NAT to confirm antibody/antigen reactivity after ART or preexposure prophylaxis (PrEP) use have been described in other studies (16, 23). Consequently, the discordant results between serology and NAT testing identified in the comparison of the CDC and alternative algorithms may reflect a general limitation of NATs in HIV diagnosis. A third test may be required when using the alternative algorithm in situations with antigen/antibody-reactive NAT-negative specimens. Further studies, including specific populations such as PrEP users, are needed to clarify the populations in which three tests would be required, if serology or NAT is optimal as the third test, as well as the frequency at which such situations arise.

The cobas HIV-1/HIV-2 Qual test was also directly compared to alternative HIV-1 and HIV-2 NATs in specimens with known discordant serology results by the CDC algorithm (e.g., Ab/Ag positive and HIV-1/HIV-2 differentiation assay negative or indeterminate). HIV-2 RNA reactivity was not observed on either assay for any serology-discordant specimen. For specimens with repeatedly reactive HIV Ag/Ab test results and negative or indeterminate Geenius assay results, the HIV-1 OPAs were 97.6% and 95.8%, respectively. Specimens such as these can indicate acute HIV infection and need to be confirmed by HIV-1 NAT and possibly another HIV-2 assay with the CDC algorithm (15). By including a NAT differentiation assay, the proposed alternative algorithm eliminates the need to resolve differentiation assay-negative or -indeterminate results while ensuring the ability to identify HIV-2 infection. However, this study was unable to determine the performance of the alternative algorithm in the setting of acute HIV infection, and future studies are needed.

It is unclear whether sample handling or other sample collection issues are another possible cause for the serology-positive, NAT-nonreactive results or if pregnancy is an independent predictor of serology-positive, HIV RNA-negative results. Although specimens with discordant results were not diluted, plasma and serum specimens used in this study were collected from 2007 to 2019 and were presumed to have been stored frozen at −20°C or below until the time of testing. However, suboptimal sample handling and storage conditions for even short periods may have compromised the specimen integrity, resulting in a degradation of RNA (24–27). The clinical performance of the proposed alternative algorithm in individuals at high risk of HIV infection may not be reflected in this study due to limitations in the study methodology, which required testing samples after prolonged storage. Notably, all 21 specimens with discordant results were from the subset of pregnant women (*n* = 236) tested from the HIV-1 high-risk group (*n* = 1,021). This observation warrants further investigation since a false-positive Geenius result was previously reported for a pregnant woman (28), and these results may also represent false-positive HIV-1 serology results. Future evaluations of the proposed alternative algorithm using prospectively collected specimens are warranted to assess the clinical performance of cobas HIV-1/HIV-2 Qual and to understand the effects of pregnancy, acute infection, ART, and PrEP on the performance of an algorithm using an HIV NAT as the HIV-1/HIV-2 differentiation assay. Evaluating the use of cobas HIV-1/HIV-2 Qual to distinguish infection from vaccine-induced seropositivity/reactivity in contemporary HIV-1 vaccine trials can also be considered (22).

The testing costs and workflow differences associated with the implementation of the proposed alternative algorithm also need to be further explored, taking into account reductions of Geenius-indeterminate results following a recent software upgrade (9), the ability of diagnostic algorithms incorporating the Architect signal-to-cutoff ratio to facilitate acute HIV diagnosis (29), and the fully automated workflow with the cobas HIV-1/HIV-2 Qual test. If our results can be generalized across laboratories, there may be appreciable gains in testing efficiency achieved by using a highly automated HIV-1/HIV-2 NAT as the differentiation assay. Two recent studies have suggested that using HIV-1 NAT after the initial HIV Ag/Ab immunoassay, rather than Geenius, can more accurately identify both acute and established infections and reduce the overall number of tests required for diagnosis (16, 30). An HIV-1/HIV-2 NAT may provide the additional advantage of HIV-2 diagnosis.

In conclusion, currently available HIV diagnostic assays used in the recommended CDC testing algorithm can create a burden on laboratories to resolve differentiation assay-negative and -indeterminate results. An alternative algorithm using the cobas HIV-1/HIV-2 qualitative NAT as the differentiation assay has good performance compared to the current CDC algorithm, and the cobas HIV-1/HIV-2 qualitative NAT has equivalent performance to alternative HIV-1 and HIV-2 NATs. The cobas HIV-1/HIV-2 qualitative NAT may be considered an alternative second step in the diagnostic algorithm for HIV type differentiation and confirmation.
