# Supplementary material for: An HIV Diagnostic Testing Algorithm Using the cobas HIV-1/HIV-2 Qualitative Assay for HIV Type Differentiation and Confirmation
Source: J Clin Microbiol. 2021 Jun 18;59(7):e03030-20. doi: 10.1128/JCM.03030-20 (PMC8218759; doi:10.1128/JCM.03030-20)
Supplement: Supplemental file 1 — Tables S1 to S9. Download JCM.03030-20-s0001.pdf, PDF file, 181 KB [file jcm.03030-20-s0001.pdf]

Supplementary material: list of Tables

Table S1. Inclusion and exclusion criteria for different groups of patients

Table S2. Overlap between Specimens Included in Agreement Analyses Between cobas HIV-1/HIV-2 Qual and Aptima Qual, UW HIV2 and/or CDC HIV Testing Algorithm

Table S3. Country of origin for specimens in Study A

Table S4. Country of origin for specimens in Study B

Table S5. Comparison Between cobas HIV-1/HIV-2 Qual and Aptima Qual NAT: HIV-1 Known Positive Specimens

Table S6. Comparison Between cobas HIV-1/HIV-2 Qual and Aptima Qual NAT: HIV-1 High Risk Specimens

Table S7. Comparison between cobas HIV-1/HIV-2 Qual and UW HIV 2 Quantitative Assay: HIV-2 Known Positive Specimens

Table S8. Comparison between cobas HIV-1/HIV-2 Qual and Comparator Assays: HIV Ab/Ag Repeat Reactive and Genius Assay Negative Specimens

Table S9. Comparison between cobas HIV-1/HIV-2 Qual and Comparator Assays: HIV Ab/Ag Repeat Reactive and Genius Assay Indeterminate Specimens

Table S1. Inclusion and exclusion criteria for different groups of patients

Group	Inclusion criteria	Exclusion criteria
HIV-1 or HIV-2 Known Positive	Known HIV-1 or -2 infection and had not been on treatment within the last month before specimen collection, OR on treatment with viral load within last 4 weeks before specimen collection greater than 100 copies/mL. Demographic and clinical information available.	Subject not HIV infected
HIV-1 High-risk	Subject had at least one of the following risk factors for HIV infection:<br>• Injection drug user<br>• Unprotected sex with an HIV-infected person, current or ever<br>• Diagnosed with sexually transmitted disease within the last year<br>• Multiple sex partners (more than one partner in the last 12 months)<br>• Was a man who had sex with men<br>• Unprotected sex with a person diagnosed with a sexually transmitted disease within the last year	Subject was known to be infected with HIV-1
HIV-2 High-risk	Subject was from an HIV-2 endemic area of West Africa and had at least one of the following risk factors for HIV infection:<br>• Injection drug user<br>• Unprotected sex with an HIV infected person<br>• Multiple sex partners (more than one partner in the last 6 months)<br>• Was a man who had sex with men	Subject was known to be infected with HIV-2
HIV Low-risk	Subject was from healthy blood donors and routine clinical visit screening in an area with less than 1% HIV prevalence	Subject was known to be infected with HIV

Table S2. Overlap between Specimens Included in Agreement Analyses Between cobas HIV-1/HIV-2 Qual and Aptima Qual, UW HIV2 and/or CDC HIV Testing Algorithm

Population	HIV-1			HIV-2		
	Study A	Study B	Overlap	Study A	Study B	Overlap
HIV-1 Known Positive	876[a]	1030	206	875[a]	n/a	n/a
HIV-2 Known Positive	145[b]	n/a	n/a	174	183	174
HIV-1 High Risk	1020[c]	519	0	1021	n/a	n/a
HIV-2 High Risk	498[d]	n/a	n/a	499	n/a	n/a
HIV Low Risk	6017	n/a	n/a	6013[e]	n/a	n/a

n/a: not applicable

[a] There was 1 HIV-1 known positive specimen was not included in the agreement analysis versus the CDC HIV Testing Algorithm for the HIV-1 target because of insufficient volume to perform HIV-1 NAT to resolve serology discordant results and 2 HIV-1 known positive specimens were not included in the agreement analysis versus the CDC HIV Testing Algorithm for the HIV-2 target because of insufficient volume to perform HIV-2 NAT to resolve serology discordant results.

[b] 29 HIV-2 known positive specimens were not included in the agreement analysis versus the CDC HIV Testing Algorithm because of insufficient volume to perform HIV-1 NAT to resolve serology discordant results.

[c] 1 HIV-1 high risk specimen was not included in the agreement analysis versus the CDC HIV Testing Algorithm because of insufficient volume to perform HIV-1 NAT to resolve serology discordant results.

[d] 1 HIV-2 high risk specimen was not included in the agreement analysis versus the CDC HIV Testing Algorithm because of insufficient volume to perform HIV-1 NAT to resolve serology discordant results.

[e] 4 HIV low risk specimens were not included in the agreement analysis versus the CDC HIV Testing Algorithm because of insufficient volume to perform HIV-2 NAT to resolve serology discordant results.

Table S3. Country of origin for specimens in Study A

Country of Origin	Study Population					Total
	**HIV-1 HIGH RISK**	**HIV-1 POSITIVE**	**HIV-2 HIGH RISK**	**HIV-2 POSITIVE**	**HIV LOW RISK**	
Cameroon	7	86		2		95
Congo		134				134
Cote d'Ivoire		16	499	117		632
Dominican Republic	50					50
France		8				8
Germany		5				5
Guinea-Bissau		17		28		45
Mexico		3				3
Peru		3				3
Senegal				9		9
South Africa		56				56
Ukraine		32				32
United States	964	321			3978	5263
Zimbabwe		1				1
Unknown		195		18	2039	2252
**Total**	1021	877	499	174	6017	8588

Table S4. Country of origin for specimens in Study B

**Country of Origin**	**HIV-1 HIGH RISK**	**HIV-1 KNOWN POSITIVE, B SUBTYPE**	**HIV-1 KNOWN POSITIVE, NON-B SUBTYPE**	**HIV-2 KNOWN POSITIVE**	**SEROLOGY DISCORDANT INDETERMINATE**	**SEROLOGY DISCORDANT NEGATIVE**	**Total**
Cameroon		21	3	2	1		**27**
Congo		28	185				**213**
Cote d'Ivoire		2	23	123	2	3	**153**
Germany		10					**10**
Guinea-Bissau			25	32			**57**
Mexico		8					**8**
Peru		2					**2**
Senegal				9			**9**
South Africa		1	41				**42**
Ukraine		5	22				**27**
United States	520	195			47	40	**914**
**Total**	**520**	**742**	**303**	**184**	**58**	**62**	**1981**

Table S5. Comparison Between cobas HIV-1/HIV-2 Qual and Aptima Qual NAT: HIV-1 Known Positive Specimens (Study B)

		Aptima Qual NAT		
**Target Analyte**	**cobas HIV-1/HIV-2 Qualitative Test**	**Positive**	**Negative**	**Total**
	**Positive**	1029	1	1030
	**Negative**	0	0	0
**HIV-1**	**Total**	1029	1	1030
	**HIV-1 PPA (95% CI)**	100%<br>(99.6% - 100%)		
	**HIV-1 NPA (95% CI)**	0%<br>(0% - 97.5%)		
	**HIV-1 OPA (95% CI)**	99.9%<br>(99.5% - 99.998%)		

Note: CI = Exact confidence interval, PPA = positive percent agreement, NPA = negative percent agreement, OPA = overall percent agreement.

Note: All specimens were ≥100 copies/mL by Alt HIV-1 Quantitative NAT.

Table S6. Comparison Between cobas HIV-1/HIV-2 Qual and Aptima Qual NAT: HIV-1 High Risk Specimens (Study B)

		Aptima Qual NAT		
Target Analyte	cobas HIV-1/HIV-2 Qualitative Test	Positive	Negative	Total
	Positive	5	0	5
	Negative	0	514	514
HIV-1	Total	5	514	519
	HIV-1 PPA (95% CI)	100%<br>(47.8% - 100%)		
	HIV-1 NPA (95% CI)	100%<br>(99.3% - 100%)		
	HIV-1 OPA (95% CI)	100%<br>(99.3% - 100%)		

Note: CI = Exact confidence interval, PPA = positive percent agreement, NPA = negative percent agreement, OPA = overall percent agreement.

Table S7. Comparison between cobas HIV-1/HIV-2 Qual and UW HIV-2 Quantitative Assay: HIV-2 Known Positive Specimens (Study B)

		UW HIV-2 Quantitative Assay		
Target Analyte	cobas HIV-1/HIV-2 Qualitative Test	Positive	Negative	Total
	Positive	182	0	182
	Negative	1	0	1
HIV-2	Total	183	0	183
	HIV-2 PPA (95% CI)	99.5%<br>(97.0% - 99.99%)		
	HIV-2 NPA (95% CI)	n/a		
	HIV-2 OPA (95% CI)	99.5%<br>(97.0% - 99.99%)		

Note: CI = Exact confidence interval, PPA = positive percent agreement, NPA = negative percent agreement, OPA = overall percent agreement, n/a = not applicable.

Note: All specimens were ≥100 copies/mL by UW HIV-2 Quantitative Assay.

Table S8. Comparison between cobas HIV-1/HIV-2 Qual and Comparator Assays: HIV Ab/Ag Repeat Reactive and Genius Assay Negative Specimens (Study B)

		Comparator Assay[a]		
Target Analyte	cobas HIV-1/HIV-2 Qualitative Test	Positive	Negative	Total
	Positive	4	0	4
	Negative	1	36	37
HIV-1	Total	5	36	41
	HIV-1 PPA (95% CI)	80.0%<br>(28.4% - 99.5%)		
	HIV-1 NPA (95% CI)	100%<br>(90.3% - 100%)		
	HIV-1 OPA (95% CI)	97.6%<br>(87.1% - 99.9%)		
		Comparator Assay[a]		
Target Analyte	cobas HIV-1/HIV-2 Qualitative Test	Positive	Negative	Total
	Positive	0	0	0
	Negative	0	41	41
HIV-2	Total	0	41	41
	HIV-2 PPA (95% CI)	n/a		
	HIV-2 NPA (95% CI)	100%<br>(91.4% - 100%)		
	HIV-2 OPA (95% CI)	100%<br>(91.4% - 100%)		

Note: CI = Exact confidence interval, PPA = positive percent agreement, NPA = negative percent agreement, OPA = overall percent agreement, n/a = not applicable.

[a] For HIV-1 target, this is Aptima Qual NAT, for HIV-2 target, this is the UW HIV-2 Quantitative Assay

Table S9. Comparison between cobas HIV-1/HIV-2 Qual and Comparator Assays: HIV Ab/Ag Repeat Reactive and Genius Assay Indeterminate Specimens (Study B)

		Comparator Assay[b]		
**Target Analyte**	**cobas HIV-1/HIV-2 Qualitative Test**	**Positive**	**Negative**	**Total**
	**Positive**	5	1	6
	**Negative**	0	18	18
**HIV-1**	**Total**	5	19	24
	**HIV-1 PPA (95% CI)**	100% (47.8% - 100%)		
	**HIV-1 NPA (95% CI)**	94.7% (74.0% - 99.9%)		
	**HIV-1 OPA (95% CI)**	95.8% (78.9% - 99.9%)		
		**Comparator Assay[b]**		
**Target Analyte**	**cobas HIV-1/HIV-2 Qualitative Test**	**Positive**	**Negative**	**Total**
	**Positive**	0	0	0
	**Negative**	0	24	24
**HIV-2**	**Total**	0	24	24
	**HIV-2 PPA (95% CI)**	n/a		
	**HIV-2 NPA (95% CI)**	100% (85.8% - 100%)		
	**HIV-2 OPA (95% CI)**	100% (85.8% - 100%)		

Note: CI = Exact confidence interval, PPA = positive percent agreement, NPA = negative percent agreement, OPA = overall percent agreement, n/a =not applicable.
[a] For HIV-1 target, this is Aptima Qual NAT, for HIV-2 target, this is the UW HIV-2 Quantitative Assay
